# Characterization and mechanisms of anti-influenza virus metabolites isolated from the Vietnamese medicinal plant *Polygonum chinense*

**DOI:** 10.1186/s12906-017-1675-6

**Published:** 2017-03-21

**Authors:** Thu Thi Tran, Meehyein Kim, Yejin Jang, Hye Won Lee, Hoa Thi Nguyen, Thanh Ngoc Nguyen, Hae Woong Park, Quang Le Dang, Jin-Cheol Kim

**Affiliations:** 1 R&D Center of Bioactive Compounds, Vietnam Institute of Industrial Chemistry, 2nd Pham Ngu Lao, Hoan Kiem, Hanoi, 10 000 Vietnam; 2 Center for Virus Research and Testing, Korea Research Institute of Chemical Technology, Gajeong-ro, Yuseong, Daejeon, 34114 Republic of Korea; 3 Department of Agricultural Chemistry, Institute of Environmentally Friendly Agriculture, College of Agriculture and Life Sciences, Chonnam National University, 77 Yongbong-Ro, Buk-Gu, Gwangju, 500-757 Republic of Korea; 4 Department of Phytochemistry, Vietnam Institute of Industrial Chemistry, 2nd Pham Ngu Lao, Hoan Kiem, Hanoi, Vietnam; 5 World Institute of Kimchi, an Annex of Korea Food Research Institute, Gwangju, 61755 Republic of Korea

**Keywords:** Antiviral, Influenza virus, *Polygonum chinense*, Ellagic acid, Methyl gallate, Caffeic acid

## Abstract

**Background:**

*Polygonum chinense* Linn. is a common medicinal plant in Southeast Asia and has been used in traditional medicine in Vietnam. The plant contains phytochemicals with various biological properties; however, its antiviral effect has not yet been demonstrated. This study was aimed to evaluate the anti-influenza virus activity of crude extracts of *P. chinense,* to characterize antiviral metabolites therefrom and to investigate their mechanisms of antiviral action.

**Methods:**

The methanol (MeOH) extract and organic solvent layers of *P. chinense* were prepared by extraction and partition with relevant solvents. The ethyl acetate (EtOAc) layer showing antiviral activity was chromatographed repeatedly on SiO_2_ and Sephadex LH-20 columns to give eight pure metabolites. Their chemical structures were determined by NMR and MS spectral data. Anti-influenza virus activity of the eight metabolites against virus strains A/Puerto Rico/8/34 (H1N1, PR8), A/Hong Kong/8/68 (H3N2, HK) and B/Lee/40 (Lee) was evaluated on the basis of cytopathic effect (CPE) and plaque inhibition assays. Time-of-addition, confocal microscopy and neuraminidase inhibition assay were performed for mode-of-action studies of active ingredients.

**Results:**

The MeOH extract of *P. chinense* showed anti-influenza virus activity with EC_50_ values ranging from 38.4 to 55.5 μg/mL in a CPE inhibition assay. Among the eight pure metabolites isolated from *P. chinense*, ellagic acid (**PC5**), methyl gallate (**PC7**) and caffeic acid (**PC8**) significantly inhibited viral replication in a dose-dependent manner in both plaque inhibition and CPE inhibition assays with EC_50_ values ranging from 14.7 to 81.1 μg/mL and CC_50_ values higher than 300 μg/mL. Mode-of-action studies suggested that **PC5** and **PC7** suppress virus entry into or replication in cells, while **PC8** targets influenza viral neuraminidase, even oseltamivir-resistant one.

**Conclusion:**

These results demonstrated that *P. chinense* and its metabolites possess effective anti-influenza virus activities. The botanical materials of *P. chinense* could be a promising multitargeted inhibitor of influenza A and B viruses and applied to development of a novel herbal medicine.

## Background

Influenza virus is an enveloped virus with negative-strand, eight-segmented RNA genomes, belonging to the *Orthomyxoviridae* family. Each viral segment is encapsidated by a virus-encoded nucleoprotein (NP), called viral ribonucleoprotein (vRNP) [1]. Influenza virions are pleomorphic, roughly spheroidal and approximately 100 nm in diameter [2]. The viral envelope is distinguished by a lipid bilayer containing three transmembrane proteins—hemagglutinin (HA), neuraminidase (NA), and matrix protein 2 (M2, ion channel) on the outside and matrix protein 1 (M1) beneath the membrane. The virus causes pandemics and annual influenza epidemics. Influenza outbreaks result in morbidity and mortality in the human population and commonly occur during winter, or the rainy season in tropical countries [3, 4].

Pharmaceutical ingredients can be classified into two groups: NA inhibitors, such as oseltamivir and zanamivir, and M2 inhibitors, such as amantadine and rimantadine. These have been approved and used to treat and prevent influenza infections. The NA inhibitors are effective against both influenza A and B viruses, while the M2 inhibitors are effective only against influenza A virus [5]. However, long-term use of these drugs is limited by their toxicity and emergence of resistance [6]. Therefore, the development of new, low-toxic anti-influenza viral drugs is required.

*Polygonum chinense* (*P. chinense*) Linn. belonging to the family Polygonaceae is a common medicinal plant found in China, India, Japan and Southeast Asian countries. In Vietnam, the plant is widespread nationwide, especially in open forest and highland [7]. This plant has been used in traditional medicine for skin infectious diseases such as eczema and zona, indigestion, gastroenteritis and hepatitis, as well as for healing inflammatory wounds or insect stings and snakebites [7, 8]. Several phytochemistry studies of *P. chinense* have been revealed that the plant contains terpenoids, alkaloids, flavonoids, tannins, steroids and glycosides [8–10]. Aqueous leaf extract of *P. chinense* possesses gastroprotective activity [11]. Its methanol (MeOH) extract showed antimicrobial, antioxidant and cytotoxic activities in vitro [8, 12]. Ethanol extract and isolated bioactive substances exerted antidiarrheal effects [13]. However, its antiviral potential has not been investigated. During screening of plant extracts against influenza viruses, we found that the methanol extract of *P. chinense* exhibited antiviral activity. Therefore, the objectives of this study were to examine the antiviral activities of its crude extracts against influenza virus strains A/Puerto Rico/8/34 (H1N1, PR8), A/Hong Kong/8/68 (H3N2, HK) and B/Lee/40 (Lee), to isolate and identify effective metabolites and to investigate their mechanisms of action.

## Methods

### Chemicals and reagents.

Silica gel 60 Å grade (particle sizes 15–40 μm and 40–63 μm) for column chromatography was purchased from Merck (Darmstadt, Germany). Sephadex LH-20 beads (size 25–100 μm) were purchased from Sigma-Aldrich (St Louis, MO). Thin-layer chromatography (TLC) plates (silica gel 60 F254, thickness 0.2 mm) were obtained from Merck. Chemical spots on TLC plates after development were detected using *p*-anisaldehyde-sulfuric acid (AS) and ferric chloride staining reagents, together with I_2_ vapor. All solvents were distillated and purified before use.

### Plant material

*P. chinense* samples were collected at Nhu Xuan in Thanh Hoa province, Vietnam. Whole plant was dried in the darkness and ground before extraction. Plant species were identified by Dr. Tran The Bach (Institute of Ecology and Biological Resources, Vietnam). A voucher specimen of the plant (No TL-CNHD.ĐT.048/13–15) was deposited in the R&D Center of Bioactive Compounds, Vietnam Institute of Industrial Chemistry, Vietnam.

### Extract preparation and isolation of pure compounds

Dried and powdered *P. chinense* (10 kg) was extracted with MeOH at room temperature and concentrated to dryness in a rotary evaporator under reduced pressure at below 40 °C. The MeOH extract (224 g) was suspended in 2 L of distillated water and consecutively partitioned with equal volumes of ethyl acetate (EtOAc) and butanol (BuOH).

The EtOAc layer (95.2 g) was separated on a Sephadex LH-20 (130 g, 70–100 μm, Sigma-Aldrich; 3.0 cm × 70 cm) with MeOH eluent. The fractions that showed similar TLC patterns were combined to yield more homogenous samples, Frs. 1 to 9. Fr. 2 (13.6 g) which contains a main component at R_f_ 0.83 on TLC (developed with EtOAc:MeOH 20:1 and showing a positive reaction with ferric chloride stain) was separated on a silica gel column [146 g silica gel 60 Å (40–63 μm), 3.4 × 40 cm] and was eluted with a dichloromethane (DCM)-acetone stepwise gradient (100: 1 to 100% acetone), yielding additional 12 fractions, Frs. 21 to 212. Fr. 26 contained a main compound and was crystalized with acetone to give crystals of **PC1**. Fr. 3 (7.2 g) was applied to a SiO_2_ column [142 g silica gel 60 Å (40–63 μm), 3.4 × 40 cm, packed with H:EtOAc (5:5, *v*/v)] and was eluted with a n-Hexane (H): EtOAc stepwise gradient (5:5 to 1:9 and EtOAc: MeOH 20:1 *v*/v, 500 mL each step) to produce five fractions, Frs. 31 to 35. Fr. 33 was further chromatographed on a silica gel column [SiO_2_ 60 Å (15–40 μm), 2.0 × 45 cm] using DCM:MeOH (9:1, *v*/v) to give Fr. 34, which contained crystals of compound **PC3**. Compound **PC4** was isolated from Fr. 35 by chromatography using silica gel [63 g, silica gel 60 Å (40–63 μm), 2.0 × 50 cm] and eluting with a H:EtOAc stepwise gradient (7:3 to 1:9, *v*/v) and mixtures of EtOAc:MeOH (50:1, 30:1, 20:1 and 8:2, 7:3, *v*/v, 150 mL each step). Fr. 31 was also isolated using a silica gel column [SiO_2_ 60 Å (15–40 μm), 2.0 × 45 cm] with a stepwise gradient elution of H:EtOAc (10:1 to 3:7; 10% gradient and 100% EtOAc), yielding compound **PC7**. A portion of Fr. 4 (1.23 g) was subjected to column chromatography using a silica gel column [37 g SiO_2_ 60 Å (40–63 μm), 3.0 × 45 cm] with eluting solvents of EtOAc: acetone (10:1, *v*/v, 200 mL) and DCM:MeOH (20:1, *v*/v, 400 mL), yielding three fractions, Frs. 41 to 43. **PC2** was crystallized from the third fraction, Fr. 43. Fr. 6 (20 g) was applied to a Sephadex LH-20 column [100 g, 70–100 μm, Sigma-Aldrich; 2.2 × 60 cm] and eluted with MeOH to give five fractions, Frs. 61–65. Fr. 63 was chromatographed using silica gel and mixtures of E:MeOH (30: and 25:1) to furnish **PC8**. Compound **PC5** was purified from Fr. 5 (4 g) using a Sep-pak C18 reverse-phase column and elution with a water-MeOH mixture with a MeOH proportion increasing from 70:30 to 100% MeOH.

A portion of Fr.1 (9.8 g) was separated on a silica gel [100 g silica gel 60 Å (40–63 μm), 4.0 × 45 cm] column and eluted using mixtures of H:acetone (98:2; 500 mL) and DCM:MeOH (95:5; 400 mL) to give seven fractions, Frs. 11 to 17. Frs. 13 and 14, which exhibited similar TLC patterns, were pooled into Fr. 134 (2.6 g) and then chromatographed on a silica gel column [70 g silica gel 60 Å (40–63 μm), 3.0 × 45 cm] with a mixture of H:acetone (8:2), yielding compound **PC6**.

### Structure determination and characterization of the isolated compounds

The chemical structures were determined by spectroscopic methods, including electrospray ionization (ESI)-mass spectroscopy (MS) and nuclear magnetic resonance (NMR) spectroscopy, and by comparison of their spectral data with values reported previously [12, 16]. The ESI-MS spectra of the purified compounds were recorded by LC-MS (Agilent 1100 Series LC/MSD Trap XCT Plus, Agilent Technologies, Palo Alto, CA). The ^1^H and ^13^C NMR, COSY, HMQC and HMBC spectra were recorded in deuterated NMR solvents using a Bruker AMX-500 FT-NMR spectrometer (Bruker Analytische Messtechnik Gmbh, Rheinstetten, Germany).

**PC1** (Quercetin): ESI-MS (positive) (*m/z*) 303 [M + H]^+^, ^1^H–NMR (500 MHz, DMSO-*d*_*6*_), *δ* (ppm): 6.183 (1H, d, *J* = 2.0 Hz, H-6), 6.403 (1H, d, *J* = 2.0 Hz, H-8), 6.88 (1H, d, *J* = 8.5 Hz, H-5′), 7.535 (1H, dd, *J* = 2.5, 8.5 Hz, H-8′), 7.67 (1H, d, *J* = 2.0 Hz, H-2′); ^13^C–NMR (125 MHz, DMSO-*d*_*6*_), *δ*(ppm): 93.308 (C8), 98.138(C6), 102.976 (C10), 115.028(C2’), 116.2 (C5′), 119.938 (C6’), 121.917(C1′), 135.683 (C3), 145.016 (C3’), 146.774 (C2), 147.662 (C4’), 156.098 (C9), 160.682 (C5), 163.837 (C7), 175.8 (C4).

**PC2** (Protocatechuic acid methyl ester): ESI-MS (negative) (*m/z)* 167.01 [M-H]^−^, ^1^H–NMR (500 MHz, acetone-*d*_*6*_), *δ* (ppm): 3.801 (3H, s, −OCH_3_), 6.893 (1H, d, *J* = 8.5 Hz, H-5); 7.435 (1H, dd, *J* = 2.0, 8.5 Hz, H-2), 7.493 (1H, d, *J* = 2.0 Hz, H-6); ^13^C–NMR (125 MHz, acetone-*d*_*6*_), *δ* (ppm): 51.84 (−OCH_3_), 115.74 (C2), 117.14 (C5), 122.83 (C1), 123.26 (C6), 145.62 (C3), 150.77 (C4), 167.07 (COO).

**PC3** (Gallic acid): ESI-MS (positive) (*m/z*) 339.0 [2 M–H]^−^, 193 [M + Na]^+^; ^1^H–NMR (500 MHz, CD_3_OD-*d*_*4*_), *δ* (ppm): 7.076 (2H, s, H-2,6); ^13^C–NMR (125 MHz, CD_3_OD -*d*_*4*_), *δ* (ppm): 110.32 (C2, 6), 122.02 (C1), 139.33 (C4), 146.38 (C3, 5), 170.49 (COOH).

**PC4** (Quercitrin): The compound was identified as quercetin 3-O-rhamnoside (also known as quercitrin) by comparison with an authentic sample (R_f_ 0.54) on a TLC plate using an E:A:M (40: 1:1, *v*/v/v) developing solvent and detected using the appropriate stains: AS and ferric chloride.

**PC5** (Ellagic acid): ESI-MS (positive) (*m/z*) 325[M + Na]^+^, ESI-MS (negative) *m/z* 301 [M-H]^−^, ^1^H–NMR (125 MHz, DMSO-*d*_6_), *δ* (ppm): 2.08–4.5 (s, −OH); 7.483 (2H, s, H-5,5′); ^13^C–NMR (500 MHz, DMSO- *d*_6_), *δ* (ppm): 107.470 (C1,1’), 110.200 (C5,5’), 112.072 (C6,6′), 136.274 (C2, 2′), 139.377 (C3, 3′), 147.920 (C4, 4′), 158.728 (C7, 7′).

**PC6** (β-Sitosterol): ^1^H–NMR (500 MHz, CDC1_3_-*d*), *δ* (ppm): 0.69 (3H, s, H-18), 0.803 (3H, d, *J* = 6.0 Hz, H-27), 0.826 (3H, d, *J* = 4.0 Hz, H-26), 0.845 (3H, t, *J* = 2.0 Hz, H-29), 0.925 (3H, d, *J* = 4.0 Hz, H-21), 1.008 (3H, s, H-19), 2.282–1.071 (28H, m), 2.305 (2H, m), 3.486 (1H, m, H-3), 5.347 (1H, brs, H-6); ^13^C–NMR (125 MHz, CDC1_3_-*d*), *δ* (ppm): 11.989 (C29), 12.215 (C24), 18.918 (C28), 19.180 (C19), 19.523 (C27), 19.945 (C26), 21.222 (C11), 23.215 (C23), 26.251 (C21), 28.376 (C16), 29.313 (C25), 31.779 (C2), 32.049 (C7), 32.049 (C8), 34.095 (C20), 36.279 (C18), 36.642(C10), 37.401 (C1), 39.923 (C12), 42.423 (C4), 42.460 (C13), 45.985 (C22), 50.286 (C9), 56.212 (C17), 56.911 (C14), 56.911 (C15), 71.908(C3), 121.817 (C6), 140.908(C5).

**PC7** (Methyl gallate): ESI-MS (positive) *m/z* 185 [M + H]^+^, ^1^H–NMR (500 MHz, CD_3_OD-*d*_6_), *δ* (ppm): 3.825 (3H, s, −OCH_3_); 7.067 (2H, s, H-2, 6); ^13^C–NMR (125 MHz, CD_3_OD-*d*_6_), *δ* (ppm): 52.250 (−OCH_3_); 110.044 (C2, 6), 121.449 (C1), 139.703 (C4), 146.441 (C3, 5), 169.007 (COO).

**PC8** (Caffeic acid): ^1^H–NMR (500 MHz, CD_3_OD-*d*_*4*_), *δ* (ppm): 6.24 (1H, d, *J* = 16 Hz, H-8), 6.80 (1H, d, *J* = 8.5 Hz, H-5), 6.94 (1H, dd, *J* = 10.5 Hz, *J* = 2.0 Hz, H-6), 7.06 (1H, d, *J* = 2.0 Hz, H-2), 7.55 (1H, d, *J* = 15.5 Hz, H-7); ^13^C–NMR (125 MHz, CD_3_OD-*d*_*4*_), *δ* (ppm): 115.104 (C2), 115.513 (C8), 116.488 (C5), 122.840 (C6), 127.803 (C1), 146.763 (C3), 147.038 (C7), 149.418 (C4), 171.022 (COOH).

### Cells, viruses and antiviral agents

Madin-Darby canine kidney (MDCK) cells and 293 T cells were purchased from ATCC (Rockville, MD) and maintained in minimum essential medium (MEM; Invitrogen, Carlsbad, CA) and in Dulbecco’s minimal essential medium (DMEM; Invitrogen), respectively, supplemented with 10% fetal bovine serum (FBS; Invitrogen) at 37 °C in 5% CO_2_. Influenza viruses PR8, HK and Lee were obtained from ATCC. Influenza A viral strains (PR8 and HK) were amplified by infection of 10-day-old chicken eggs at 37 °C for 3 days and influenza B virus (Lee) by infection of MDCK cells under serum-free conditions. Viruses were partially purified from harvested egg allantoic fluid or cell culture supernatant by centrifugation at 3000 rpm for 5 min. Viral titers were determined by HA assay or plaque assay, and stocks were stored at −70 °C before use. Antiviral agents, ribavirin (RBV) (Sigma-Aldrich) and oseltamivir carboxylate (OSV-C; US Biological, Swampscott, MA) were used as control compounds. The test extracts and purified compounds, with the exception of the water layer which was dissolved in water, were dissolved in DMSO to 40 mg/mL and stored at −20 °C.

### Rescue of PR8 virus with the H275Y mutation in NA

DNAs derived from eight-segmented PR8 genomic RNAs were individually cloned into a vector harboring convergent RNA polymerase I and II promoters using a universal primer and genome-specific primers, generating pVP-PB2, −PB1, −PA, −HA, −NP, −NA, −M and -NS [17–19]. Their sequences were compared to those in GenBank (accession numbers AB671295 for PB2, KC866596.1 for PB1, KC866595.1 for PA, AB671289.1 for HA, KC866598.1 for NP, CY033579.1 for NA, EF467824.1 for M and EF467817 for NS, respectively). Using the plasmid pVP-NA, a histidin-to-tyrosin mutation at amino acid 275 (H275Y) was introduced with a primer (5′-GAATGCACCTAATTTTTAC**TAC**GAGGAATGTTC-3′: sequences corresponding to the mutant tyrosin in bold) and its complementary one. The QuickChange II Site-Directed Mutagenesis Kit (Stratagene, LA Jolla, CA) were used to generate pVP-NA(H275Y). For production of PR8 virus with the NA mutation H275Y [rgPR8(H275Y)] by a reverse genetics system, 293 T cells were co-trasfected with pVP-NA(H275Y) and the rest seven pVP plasmids [17]. The rescued virus from the culture supernatants was propagated into embryonated chicken eggs and viral titer was determined as mentioned above.

### Cytopathic effect (CPE) inhibition assay

Cell viability was measured according to our previous report [20]. In brief, MDCK cells were seeded in 96-well plates and grown until 100% confluence. Cells were mock-infected or infected with influenza virus at a multiplicity of infection (MOI) of 0.001 for 1 h at 35 °C under serum-free conditions. Unabsorbed virus was removed by washing with PBS, and threefold serial dilutions of compounds dissolved in MEM with 2 μg/mL TPCK-trypsin were added to each well. On day 3 postinfection (p.i.), cell viability was measured using 3-(4,5-dimethylthiazol-2-yl)-2,5-diphenyltetrazolium bromide (MTT; Sigma-Aldrich). After reading the absorbance at 540/690 nm, the half maximal cytotoxic concentration (CC_50_) and the half maximal effective concentration (EC_50_) were calculated using GraphPad Prism 6 (GraphPad Software, La Jolla, CA). The selectivity index (S.I.) is the ratio of CC_50_ to EC_50_.

### Plaque inhibition assay

MDCK cells seeded in 48-well plates were infected with the influenza viruses at an MOI of 0.001 for 1 h at 33 °C (PR8) or 35 °C (HK and Lee). Test compounds and reference antiviral compounds were serially diluted in overlay medium [MEM containing 0.5% carboxymethylcellulose (CMC; Sigma) and 2 μg/mL TPCK-trypsin]. After washing with PBS, the cells were treated with the compounds in the overlay medium for 3 days at the temperature used for virus infection. Viral plaques were counted by staining with crystal violet [20].

### Time-of-addition

To determine when the purified materials, **PC5**, **PC7** and **PC8**, express their anti-influenza viral activity, MDCK cells were treated with increasing concentrations (from 4 to 100 μM) of each compound for 2 h before (pre-treatment), during (co-treatment) or after (post-treatment) PR8 infection [40 plaque forming units (PFU) per well in 48-well plates] at 37 °C. After washing with PBS, cells were incubated with overlay medium for 3 days at 33 °C and stained with crystal violet to count the number of viral plaques.

### Confocal microscopy

Confluently grown MDCK cells in 4-well slides were infected with the influenza viruses at an MOI of 1 for 4 h in the presence either of 1 mM **PC5**, **PC7** or **PC8**, or of 10 μM epigallocatechin gallate (EGCG), which was used as a control to block viral entry [19]. After fixation and permeabilization, cells were blocked with 10% goat serum and 1% bovine serum albumin (BSA; Sigma-Aldrich) in PBS. Viral NP was detected with mouse anti-NP antibody (Santa Cruz Biotechnology, Santa Cruz, CA) and Alex Fluor 488-conjugated goat anti-mouse IgG (Invitrogen). Nuclei were stained with DAPI (Vector Laboratories, Burlingame, CA) according to the manufacturer’s instructions.

### NA inhibition assay

Twenty-five microliters of PR8 or rgPR8(H275Y) (2 × 10^5^ PFU/mL) in PBS were mixed with an equal volume of 3-fold serially diluted **PC5**, **PC7**, **PC8** (from 6 mM to 80 μM) or OSV-C (from 60 to 0.8 nM) in PBS in 96-well plates. After addition of 50 μL 200 μM NA-fluor fluorescent substrate (Applied Biosystems, Foster City, CA), the reaction was incubated at 37 °C for 1 h and stopped with 100 μL of stop solution. The fluorescent intensity was measured with excitation at 350 nm and emission at 450 nm.

### Statistical analysis

Statistically significant differences were determined using two-tailed Student’s *t*-test. *P* < 0.05 was considered significant.

## Results and discussion

### *Screening of antiviral activities in extracts from P. chinense*

The anti-influenza viral activity of MeOH extract and organic solvent layers from *P. chinense* was evaluated by CPE inhibition assay. Cell viability was measured using MTT to estimate the CC_50_ and EC_50_ values before and after infection of MDCK cells with PR8, HK and Lee (Table 1). As expected, OSV-C (an NA inhibitor) and RBV (a viral polymerase inhibitor) efficiently inhibited the replication of all viral strains tested, confirming the reliability of the assay. MeOH extract of *P. chinense* showed moderate inhibition of influenza viruses: EC_50_ values were 55.0 μg/mL for PR8, 38.8 μg/mL for HK and 55.5 μg/mL for Lee. The EtOAc layer derived from the MeOH extract by liquid-liquid partitioning exhibited EC_50_ values of 46.9, 23.2 and 50.8 μm/mL, respectively. Moreover, the MeOH extract and EtOAc layer were not cytotoxic to MDCK cells at the highest concentration used, 300.0 μg/mL. The BuOH layer also displayed antiviral efficacy similar to EtOAc layer. However, the aqueous layer showed limited or no activity.

**Table 1:** Antiviral activity of the crude extracts of *P. chinense* against influenza viruses in the CPE reduction assay

Sample	CC_50_^a^	EC_50_^b^ (S.I.^c^)			Unit
		PR8^d^	HK^e^	Lee^f^	
MeOH extract	> 300.0	55.0 (> 5.5)	38.4 (> 7.8)	55.5 (> 5.4)	μg/mL
Water layer	> 300.0	175.8 (>1.7)	68.5 (> 4.4)	212.1 (> 1.4)	μg/mL
BuOH layer	> 300.0	45.9 (> 6.5)	18.3 (> 16.4)	70.1 (> 4.3)	μg/mL
EtOAc layer	> 300.0	46.9 (> 6.4)	23.2 (> 13.0)	50.8 (> 5.9)	μg/mL
OSV-C^g^	> 100.0	0.38 (> 263.2)	0.005 (> 20,000)	1.2 (> 83.3)	μM
RBV^h^	> 100.0	49.9 (> 2.0)	20.5 (> 4.9)	26.6 (>3.8)	μM

^a^CC_50,_ 50% cell toxicity concentration; ^b^EC_50_, 50% effective concentration; ^c^S.I., selectivity index = CC_50_/EC_50_; ^d^PR8, A/Puerto Rico/8/34 (H1N1); ^e^HK, A/Hong Kong/8/68 (H3N2); ^f^Lee, B/Lee/40; ^g^OSV-C, oseltamivir carboxylate; ^h^RBV, ribavirin

### *Isolation and identification of substances from P. chinense*

**Fig. 1:**
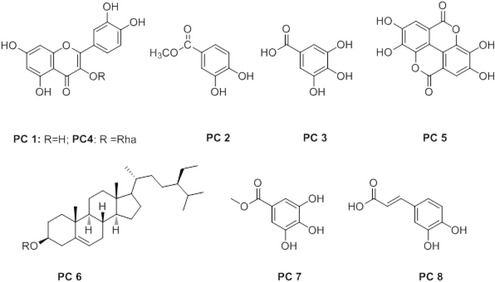
Chemical structures of substances isolated from *P. chinense*. **PC1**: quercetin; **PC2**: protocatechuic acid methyl ester; **PC3**: gallic acid; **PC4**: quercitrin; **PC5**: ellagic acid; **PC6**: β-sitosterol; **PC7**: methyl gallate; and **PC8**: caffeic acid

Using repeated chromatographic processes, eight substances, **PC1** to **PC8**, were further isolated from the EtOAc layer (Fig. 1). Compound **PC1** was purified as yellow crystals from Fr. 2. The ESI-MS (positive) spectrum of **PC1** showed a pseudo-molecular ion peak [M + H]^+^ at *m/z* 303; this suggested it to have a molecular formula of C_15_H_10_O_7_. The 1D–NMR spectrum of **PC1** showed five ^1^H–NMR peaks, presenting aromatic proton signals in the down-field region and 15 carbon signals with characteristics of a C6-C3-C6 structure in the ^13^C–NMR spectrum. By comparison of the 1D–NMR data of **PC1** with those in the literature, **PC1** was identified as quercetin [15].

Compounds **PC2**, **PC3, PC5** and **PC7** were determined from their ^13^C–NMR spectra as derivatives of gallic acid with six aromatic carbon signals and a carbonyl group. The molecular weight and formula of **PC3** were determined to be 170 and C_7_H_6_O_5_, respectively (339.0 [2 M–H]^−^in ESI-MS-negative; 193 [M + Na]^+^ in ESI-MS-positive). Compound **PC3** was identical to gallic acid on the basis of 1D–NMR data analysis and comparison with those in the literature [16]. Compound **PC7** was identified as methyl gallate, which has a molecular formula of C_8_H_8_O_5_ (*m/z* 185 [M + H]^+^ in ESI-MS) and showed patterns of proton and carbon signals similar to those of gallic acid (**PC3**), with the exception of signals at 3.825 ppm (3H, s, −OCH_3_) in ^1^H–NMR and 52.250 ppm (−OCH_3_) in ^13^C–NMR, suggesting a methoxy group. Compound **PC2** was identical to protocatechuic acid methyl ester, and was obtained as white crystals. Its molecular formula was deduced to be C_8_H_8_O_4_ from a [M-H]^−^ pseudo-molecular ion peak at *m/z* 167.01 in ESI-MS. The ^13^C–NMR spectrum of **PC2** was similar to that of **PC7**; it also revealed the presence of eight carbon signals, comprising a carbonyl group at 167.07 ppm, a methoxy at 51.84 ppm and six aromatic carbons from 115.74 to 150.77 ppm including three methine groups at 115.74, 117.14 and 123.26 ppm. In comparison with the ^1^H–NMR data of **PC7**, **PC2** revealed a further two aromatic methines. Moreover, the ^1^H–NMR spectrum of **PC2** presented three aromatic protons at 7.435 (1H, dd, *J* = 2.0, 8.5 Hz, H-2), 6.893 (1H, d, *J* = 8.5 Hz, H-5), 7.493 (1H,d, *J* = 2.0 Hz, H-6), and a methoxy group at 3.801 (3H, s, −OCH_3_), which corresponds to a methyl ester bond based on a cross-peak at 3.801 ppm and 167.07 ppm in HMBC. **PC5** was identified as ellagic acid and was obtained as an amorphous solid with the structural formula C_14_H_6_O_8_ based on pseudo-molecular ion peaks at *m/z* 325 [M + Na]^+^ in ESI-MS (positive mode) and *m/z* 301 [M-H]^−^ in ESI-MS (negative mode). The chemical structure of **PC5** was determined by comparison with ^1^H- and ^13^C–NMR data published previously [12].

Compound **PC8** was purified from Fr. 6 as an amorphous solid. Its chemical structure was identical to that of caffeic acid on the basis of ^1^H- and ^13^C–NMR data analysis and comparison of these data with those in the literature [13]. Compounds **PC4** and **PC6** were identified by comparing their R_f_ values with those of authentic compounds that were spotted and developed on the same TLC. **PC4** was identified as quercitrin (quercetin 3-rhamnoside) and contained a minor gallic acid impurity. Therefore, this compound was not tested in our bioassays. The ^1^H- and ^13^C–NMR spectra of **PC6** were compared with those reported previously [14]. Among eight compounds isolated from *P. chinense*, **PC6** was identified as β-sitosterol, while the others were related to flavonoid and phenolic metabolites found commonly in the Polygonaceae family [10, 13, 15]. All **PC** compounds, except for **PC4,** have been reported to possess antifungal, antioxidant and other pharmaceutical properties [13, 16]. In this study, it was aimed to investigate anti-influenza virus activities of the *P. chinense* constituents systematically and their mode-of-action.

### Anti-influenza virus activity of isolated compounds

**Fig. 2:**
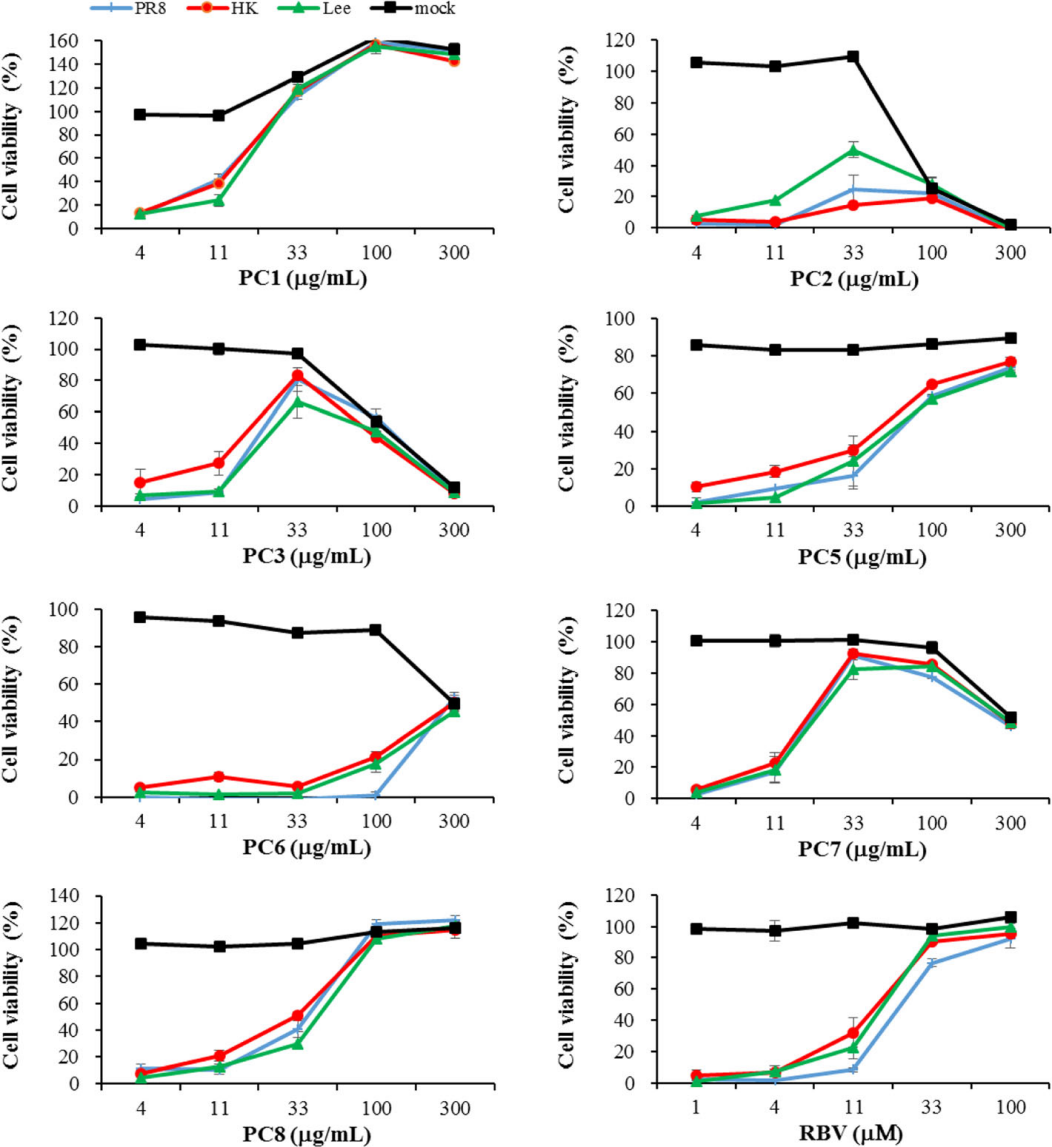
Dose response curves of **PC1** to **PC3** and **PC5** to **PC8** isolated from *P. chinense* against PR8, HK and Lee and the MDCK host cell line. Cell monolayers were mock-infected or infected with influenza A and B viruses at a MOI of 0.001 for 1 h. After washing with PBS, compounds serially diluted in MEM with 2 μg/mL TPCK-trypsin were added to the wells and incubated for 3 days. Cell viability was measured by MTT assay. RBV was used as a positive control. The values are means ± standard deviation (SD) from three replicates

To study the in vitro anti-influenza virus effects of the isolated substances, CPE inhibition assay was performed (Table 2 and Fig. 2). As reported in previous studies [21, 22], **PC1** showed anti-influenza A virus activity with an EC_50_ of 12.6 μg/mL and 13.1 μg/mL for PR8 and HK, respectively, without cytotoxicity at 300 μg/mL (Table 2). This result was in agreement with the report by Wu et al. [22], in which quercetin revealed broad-spectrum inhibitory activity against influenza A viruses such as PR8, A/FM-1/47/1 (H1N1) and A/Aichi/2/68 (H3N2) by interaction with the viral HA2 subunit. However, in our study, the compound induced overgrowth of uninfected MDCK cells, resulting in 150–160% increases in cell viability at 100 and 300 μg/mL (Fig. 2). This undesirably increased optical density was likely not due to the colorimetric properties of **PC1**, as it was not detected in MTT-based viability tests of other cells, such as Vero (African green monkey kidney cell line), MT4 (human T-cell line) or HuT 78 (human T-cell lymphoma cell line), in the presence of **PC1** (data not shown). It was expected that **PC1** non-specifically stimulated cell viability of MDCK cells. **PC6** displayed marginal or no anti-influenza activity and cytotoxicity to MDCK cells, with a CC_50_ value of 300 μg/mL (Table 2 and Fig. 2). Accordingly, **PC1** and **PC6** were excluded from further antiviral assays.

**Table 2:** Antiviral activity of substances isolated from *P. chinense* against influenza viruses in the CPE reduction assay

Sample	Identity	CC_50_^a^	EC_50_^b^ (S.I.^c^)			Unit
			PR8^d^	HK^e^	Lee^f^	
PC1	quercetin	> 300.0	12.6 (> 23.8)	13.1 (> 22.9)	15.0 (> 20.0)	μg/mL
PC2	protocatechuic acid methyl ester	24.1	> 24.1 (N.A.^g^)	> 24.1 (N.A.)	11.1 (2.2)	μg/mL
PC3	gallic acid	111.1	20.8 (5.3)	17.5 (6.3)	24.5 (4.5)	μg/mL
PC5	ellagic acid	> 300.0	81.1 (> 3.7)	62.4 (> 4.8)	79.2 (> 3.8)	μg/mL
PC6	β-sitosterol	300.0	281.6 (1.1)	300.0 (1.0)	> 300.0 (N.A.)	μg/mL
PC7	methyl gallate	> 300.0	18.1 (> 16.6)	17.1 (> 17.5)	19.2 (> 15.6)	μg/mL
PC8	caffeic acid	> 300.0	37.8 (> 7.9)	32.1 (> 9.4)	14.7 (> 20.4)	μg/mL
RBV^h^	-	> 100.0	21.7 (> 4.6)	15.5 (> 6.5)	17.0 (> 5.9)	μM
OSV-C^i^	-	> 100.0	0.07 (> 1428)	< 0.005 (> 20,000)	0.21 (> 476)	μM

^a^CC_50,_ 50% cell toxicity concentration; ^b^EC_50_, 50% effective concentration; ^c^S.I., selectivity index = CC_50_/EC_50_; ^d^PR8, A/Puerto Rico/8/34 (H1N1); ^e^HK, A/Hong Kong/8/68 (H3N2); ^f^Lee, B/Lee/40; ^g^ *N.A.* not applicable; ^h^ *OSV-C* oseltamivir carboxylate; ^i^ *RBV* ribavirin

Compounds **PC2**, **PC3**, **PC5** and **PC7** are gallic acid and its derivatives (Fig. 1), all of which, except for **PC2**, exhibited activity against influenza A and B strains (Table 2). In terms of selectivity, **PC7** showed the most desirable antiviral profile with EC_50_ values of 18.1, 17.1 and 19.2 μg/mL against PR8, HK and Lee, respectively. However, it also contained latent cytotoxicity, reducing cell viability by 49% at the maximum concentration, 300 μg/mL (Fig. 2). The inhibition of PR8 by **PC7** was described in a previous report [23]. Nevertheless, it is noteworthy that this may be the first report of the anti-influenza activity of methyl gallate (**PC7**) against additional strains A/H3N2 and B. Consistent with other researchers’ observation [21, 24], we found that gallic acid (**PC3**) and ellagic acid (**PC5**) are active against influenza viruses, even though **PC3** showed cytotoxicity with CC_50_ of 111.1 μg/mL (Table 2). The anti-influenza viral activity of gallic acid and its derivatives as well as quercetin could be associated with their planar structure and cell membrane permeability, as described previously [21]. The last compound, **PC8** showed desirable antiviral activity with EC_50_ values ranging from 14.7 to 37.8 μg/mL without cytotoxicity or an abnormally increase in cell viability at the maximum concentration treated (300.0 μg/mL) (Table 2 and Fig. 2).

**Fig. 3:**
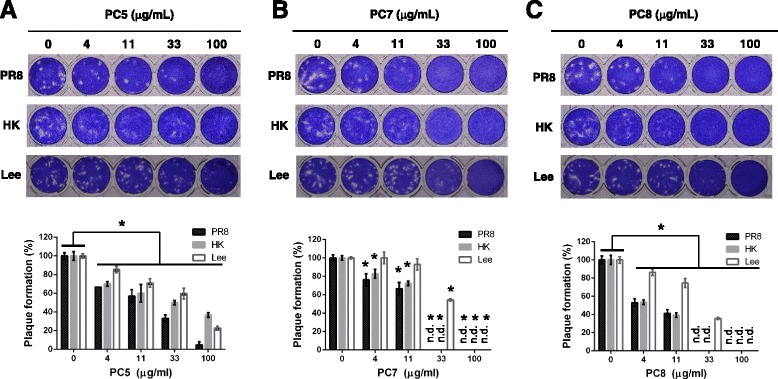
Plaque inhibition assay of **PC5** (**a**), **PC7** (**b**) and **PC8** (**c**) against PR8, HK and Lee. At 1 h after infection with influenza virus at an MOI of 0.001, MDCK cells were treated with serially diluted compounds dissolved in the overlay medium. At day 3 postinfection, viral plaques were visualized using crystal violet staining. The images are representative of three experiments (*upper*). The numbers of plaques in each well were counted (lower). Shown are averaged percentages and SD of three different wells. n.d., not detected. *P < 0.05

The antiviral activity of the three most effective compounds, **PC5**, **PC7** and **PC8**, was compared by plaque inhibition assay, a standard method for measuring the activity of viral inhibitors. Consistent with results from the CPE assay (Table 2), they inhibited plaque formation of influenza A and B viruses in a dose-dependent manner (Fig. 3). Our findings suggest that among the three compounds, **PC8** has the greatest antiviral efficacy and lowest cytotoxicity. Even though **PC5** suppressed plaque formation significantly at all concentrations tested (*P* < 0.05), it was less potent than **PC7 and PC8** (Fig. 3a). **PC7** showed potent antiviral activity with relatively high S.I. values (Table 2), but it seemed to have marginal cytotoxicity given that crystal violet staining resulted in faint background images at concentrations higher than 33 μg/mL (Fig. 3b). This observation was in agreement with the CPE assay graph (compare Figs. 2 and 3), confirming the reliability of the cell culture-based antiviral screening system. The antiviral activity of caffeic acid (**PC8**) against influenza A virus, including H1N1 and H3N2 strains, was reported previously [25]. Herein, we evaluated the spectrum of its efficacy also against influenza B virus (Fig. 3c).

### Mode-of-action of **PC5**, **PC7** and **PC8**

**Fig. 4:**
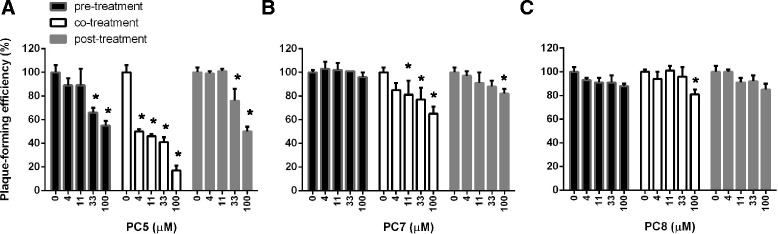
Time-of-addition of the **PC** compounds. Mock (0.1% DMSO) or increasing concentrations of **PC5** (**a**), **PC7** (**b**) or **PC8** (**c**) were applied to MDCK cells for 2 h before (pre-treatment), during (co-treatment) or after (post-treatment) PR8 infection (40 PFU per well in 48-well plates) at 37 °C. Cells were washed with PBS and additionally incubated under the overlay medium for 3 days at 33 °C. The number of plaques was counted after staining with crystal violet. The values shown are the means ± S.D. of triplicate samples and expressed as a percentage relative to the plaque number with mock solution in each experiment. *P < 0.05

**Fig. 5:**
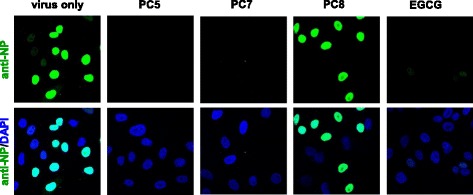
Confocal microscopy. MDCK cells were infected with PR8 virus at an MOI of 1 in the presence either of 1 mM **PC5**, **PC7** or **PC8** or of 10 μM EGCG (an entry blocker) at 37 °C for 4 h. The viral NP was labeled with mouse anti-NP antibody and Alexa Fluor 488-conjugated goat anti-mouse antibody (green). Cellular nuclei were counterstained with DAPI (blue). Original magnification, 400 ×

Although anti-influenza viral activity of ellagic acid (**PC5**), methyl gallate (**PC7**) and caffeic acid (**PC8**) in vitro or in vivo was presented in several previous reports [23, 25, 26], their mechanisms of action have not yet been elucidated clearly except for **PC8**, that targets viral NA protein [27]. To investigate their effects on virus entry and early replication in cells, time-of-addition experiment was performed (Fig. 4). Treatment of **PC5** for 2 h before (pre-treatment) or after (post-treatment) reduced viral plaque titers, while plaque reduction was more distinct when it was treated during virus infection (co-treatment) (Fig. 4a). It means that **PC5** could affect both viral and cellular factors working at early steps of the virus life cycle and potentially interfere with their interaction. **PC7** also showed the most significant antiviral activity when it was co-treated, resulting in about 35% reduction (Fig. 4b). It marginally reduced the viral infectivity by 15% at the maximum concentration (100 μM) in the post-treatment experiment, while no inhibition when treated before virus infection. It is expected that **PC7** might regulate a host factor essential for influenza virus entry or early replication. **PC8** resulted in no significant antiviral activity when treated before or after virus infection, but its co-treatment barely showed the efficacy at the maximum concentration (Fig. 4c). It can be suggested that **PC8** is partially involved in suppression of the influenza virus entry step. To ensure that **PC5** and **PC7** suppress influenza virus entry or early replication, we analyzed viral NP protein production in cells by confocal microscopy (Fig. 5). Here, the 4-h incubation of influenza A virus PR8 was optimized for nuclear localization of NP. The result clearly showed that both **PC5** and **PC7** inhibit the early steps similar to EGCG, which is a virus entry blocker [20]. As expected, **PC8** did not affect NP signal intensity or its localization within 4 h after virus infection.

**Fig. 6:**
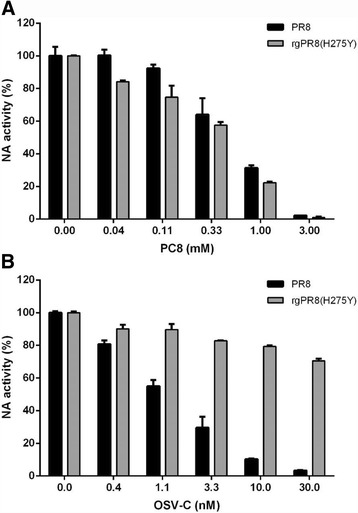
NA inhibition assay. The NA activity of influenza virus PR8 and rgPR8(H275Y) was measured in the presence of increasing concentrations of **PC8** (**a**) or OSV-C (**b**). After addition of the NA-Fluor fluorescent substrate, enzymatic activity was measured by reading the fluorescence intensity with excitation at 350 nm and emission at 450 nm. The data shown are the means ± S.D. of three different samples

To further examine whether **PC8**, known as an NA inhibitor [27], targets not only wild-type NA protein but also its OSV-C-resistant one, fluorescence NA inhibition assay was performed using live influenza viruses, PR8 and rgPR8(H275Y) (Fig. 6). Although NA inhibitory effect of **PC8** against PR8 was less potent than that of OSV-C, it showed consistent activity against rgPR8(H275Y), that is a reverse genetically rescued virus resistant to OSV-C (compare panels A and B in Fig. 6). It suggested that **PC8** targets influenza virus NA protein as OSV-C does but in a different binding manner. Moreover, previous reports proposed that the NA activity is involved in influenza virus entry as well as release of progeny virus [28]. Thus, it can be explained the reason for marginal inhibition of the initial stage of PR8 infection by the NA inhibitor, **PC8** (Fig. 4c).

## Conclusion

The present study is the first report on anti-influenza virus activity of *P. chinense* and identification of the active components. MeOH extract, EtOAc and BuOH layers of the plant were active against influenza viruses of A/H1N1, A/H3N2 and B. Among constituents isolated from the EtOAc layer, ellagic acid, methyl gallate and caffeic acid exhibited considerable inhibitory activity against the pathogens. Based on the mode-of-action studies, ellagic acid and methyl gallate suppress the early steps of the viral life cycle. In contrast caffeic acid targets the NA protein, comparably suppressing both wild-type and OSV-C-resistant influenza viruses. Our study suggests that the multi-functional botanical materials of *P. chinense* could be promising inhibitors of influenza A and B viruses and applied to development of a novel herbal medicine.

## Abbreviations

BuOH: Butanol
CC_50_: The half maximal cytotoxic concentration
CMC: Carboxymethylcellulose
CPE: Cytopathic effect
EC_50_: The half maximal effective concentration
ESI-MS: Electrospray ionization-mass spectroscopy
EtOAc: Ethyl acetate
FBS: Fetal bovine serum
HA: Hemagglutinin
HK: Virus strain A/Hong Kong/8/68 (H3N2)
Lee: Virus strain B/Lee/40
M2: Matrix protein 2
MDCK: Madin-Darby canine kidney
MEM: Minimum essential medium
MeOH: Methanol
MOI: Multiplicity of infection
MTT: 3-(4,5-dimethylthiazol-2-yl)-2,5-diphenyltetrazolium bromide
NA: Neuraminidase
NMR: Nuclear magnetic resonance
OSV-C: Oseltamivir carboxylate
PR8: Virus strain A/Puerto Rico/8/34 (H1N1)
RBV: Ribavirin
S.I.: Selectivity index
TLC: Thin-layer chromatography
